# Insights into Intra-Tumoral Heterogeneity: Transcriptional Profiling of Chemoresistant MPM Cell Subpopulations Reveals Involvement of NFkB and DNA Repair Pathways and Contributes a Prognostic Signature

**DOI:** 10.3390/ijms222112071

**Published:** 2021-11-08

**Authors:** Mario Cioce, Andrea Sacconi, Harvey I. Pass, Claudia Canino, Sabrina Strano, Giovanni Blandino, Vito Michele Fazio

**Affiliations:** 1 Department of Medicine, R.U. in Molecular Medicine and Biotechnology, University Campus Bio-Medico of Rome, 00128 Rome, Italy; 2 Clinical Trial Center, Biostatistics and Bioinformatics Unit, IRCCS Regina Elena National Cancer Institute, 00144 Rome, Italy; andrea.sacconi@ifo.gov.it; 3 Division of General Thoracic Surgery, Department of Cardiothoracic Surgery, New York University Langone Medical Center, New York, NY 10016, USA; Harvey.Pass@nyulangone.org; 4 Radiation Oncology Unit, UPMC Hillmann Cancer Center, San Pietro Hospital FBF, 00189 Rome, Italy; cldcanino@gmail.com; 5 Small Animal Facility Unit, Department of Research, Diagnosis and Innovative Technologies, IRCCS Regina Elena National Cancer Institute, 00144 Rome, Italy; sabrina.strano@ifo.gov.it; 6 Oncogenomic and Epigenetic Unit, IRCCS Regina Elena National Cancer Institute, 00144 Rome, Italy; giovanni.blandino@ifo.gov.it; 7 Laboratory of Oncology, Institute of Translational Pharmacology, National Research Council of Italy (CNR), 00133 Rome, Italy; 8 Fondazione IRCCS Casa Sollievo della Sofferenza, 71013 San Giovanni Rotondo, Italy

**Keywords:** chemoresistance, ALDH, gene expression, NFkB, DNA repair, butein, mesothelioma, intra-tumoral heterogeneity (ITH), Cancer Stem Cell (CSC)

## Abstract

Chemoresistance is a hallmark of malignant pleural mesothelioma (MPM) management and the expression of ALDH1A3 is responsible for the survival and activity of MPM chemoresistant cell subpopulations (ALDH^bright^ cells). We enriched mesothelioma ALDH^bright^ cells to near homogeneity by FACS sorting and an Aldefluor assay and performed unbiased Affymetrix gene expression profiling. Viability and ELISA assays were used to rule out significant apoptosis in the sorted cell subpopulations and to assess target engagement by butein. Statistical analysis of the results, pathway enrichment and promoter enrichment were employed for the generation of the data. Q-RTPCR was used to validate a subset of the identified, modulated mRNAs In this work, we started from the observation that the mRNA levels of the ALDH1A3 isoform could prognostically stratify MPM patients. Thus, we purified MPM ALDH^bright^ cells from NCI-H2595 cells and interrogated their gene expression (GES) profile. We analyzed the GES of the purified cells at both a steady state and upon treatment with butein (a multifunctional tetrahydroxy-chalcone), which abates the ALDH^bright^ cell number, thereby exerting chemo-sensitizing effects in vitro and in vivo. We identified 924 genes modulated in a statistically significant manner as a function of ALDH status and of the response to the inhibitor. Pathway and promoter enrichment identified the molecular determinant of high ALDH status and how butein treatment altered the molecular portrait of those chemoresistant cell subpopulations. Further, we unraveled an eighteen-gene signature with high prognostic significance for MPM patients, and showed that most of the identified prognostic contributors escaped the analysis of unfractionated samples. This work proves that digging into the unexplored field of intra-tumor heterogeneity (ITH) by working at the cell subpopulation level may provide findings of prognostic relevance, in addition to mechanistic insights into tumor resistance to therapy.

## 1. Introduction

Resistance to chemotherapy involves multiple genes and multiple mechanisms, including a rearrangement of cell populations endowed with an adaptive ability to therapy-induced stress [1,2,3,4]. Despite important progress at identifying mediators of resistance at the genetic and epigenetic level [5,6,7], what mediates the dynamic remodeling of cell subpopulations within drug-challenged tumors remains relatively unexplored. This falls within a more general scenario through which intra-tumor heterogeneity (ITH) fuels cancer resistance to therapy [8,9], allowing the tumor and its microenvironment to function as a complex ecosystem [10]. Malignant pleural mesothelioma (MPM) is an inflammation-driven neoplastic disease of the parietal pleura lining the lungs. MPM represents an important therapeutic challenge for oncologists [11]. Despite some very recent success with immune checkpoint inhibitors [12,13], the currently employed therapies provide only a subtle survival advantage to late-stage or inoperable patients [14]. In fact, the median survival time of the patients from the diagnosis is 8–14 months [15]. This is due to both the silent clinical course of the disease and to a strong chemoresistance of the MPM cells, the latter of which is demonstrated in vitro and ex vivo [16]. We and others have shown that pemetrexed and cisplatin treatment of cell lines and primary cultures triggered the emergence of cell subpopulations exhibiting absolute chemoresistance, mesenchymal traits and high levels of aldehyde dehydrogenase (ALDH) activity (ALDH^bright^ cells and those properties were shared also by other tumors such as ovarian and lung cancer ALDH^bright^ cells [17,18,19]. ALDHs are a family of enzymes with heterogeneous intracellular localization and substrate specificity [20], which function by oxidizing intracellular aldehydes to carboxylic acid in physiological and patho-physiological conditions [21]. ALDH^bright^ cells represent, quantitatively, the main chemoresistant cell subpopulation in several tumors in a conspicuous number of developmentally unrelated tumors [22,23]. Both in vitro and ex vivo, we and others have shown that the ALDH activity is largely responsible for the chemoresistance of MPM cells, and MPM ALDH^bright^ cells are enriched for the expression of the ALDH1A3 isoform. ALDH1A3 expression in MPM was under the control of an NFkB-STAT3-DDIT3 axis in chemotherapy-challenged cultures [24]. Thus, the ALDH status may relate to cellular functions including self-renewal and resistance to drugs and radiation [25]. In this work, we started from the observation that expression of the ALDH1A3 isoform in MPM can prognostically stratify patients in terms of overall survival. We purified, to near homogeneity, MPM ALDH^bright^ cells from NCI-H2595 cells and interrogated their gene expression profile. We analyzed the purified cells at both a steady state and upon treatment with butein (a multifunctional tetrahydroxy-chalcone), which we and others have shown to abate the ALDH^bright^ cell number, thereby exerting chemo-sensitizing effects in vitro and in vivo [24,26]. We collected the gene expression data from the purified cell subpopulations treated with butein, in non-apoptotic conditions, to avoid potential confounding effects of apoptosis-related processes. Pathway- and promoter-enrichment analysis strengthen the relevance of the NFkB pathway in mediating the survival of the ALDH^bright^ cells and have shown how butein treatment modulates DNA damage and proliferation-associated pathways, thereby supporting the observed chemo-sensitizing effects of the drug when co-administered with chemotherapy, at least partially through NFkB modulation [24,27,28,29,30,31,32,33]. Finally, by taking into consideration both mRNAs enriched in the ALDH^bright^ cells and those more deeply downregulated in the latter cells by butein treatment, we identified an eighteen-gene signature that held prognostic potential in 84 MPM patients from the TGCA database.

## 2. Results

### 2.1. ALDH1A3 Expression May Stratify MPM Patients

**Figure 1:**
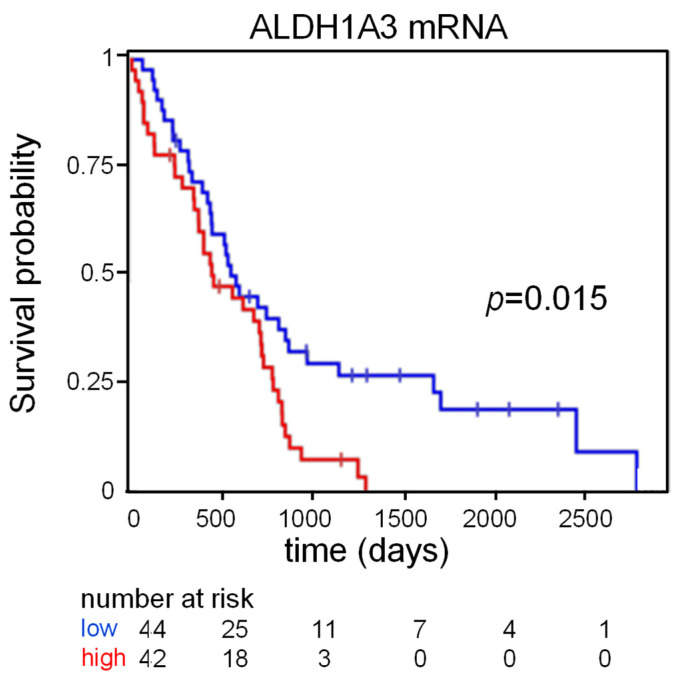
**Expression of ALDH1A3 holds prognostic significance in MPM:** Kaplan–Meier plot from TGCA samples (*n* = 84) showing stratification of MPM patients based on ALDH1A3 mRNA levels. Overall survival (days) is shown on the X-axis. *p*-value is reported.

We showed that ALDH1A3 mRNA is enriched in ALDH^bright^ cells [24] and that expression of ALDH1A3 accounted for most of the ALDH activity detected in the MPM samples [24]. On this basis, we evaluated the possibility that expression of the ALDH1A3 mRNA may stratify the MPM patients in a prognostically relevant way. Analysis of 84 MPM patients from the TGCA database revealed that this was the case (Figure 1) (*p* = 0.014). Thus, ALDH1A3 mRNA levels could discriminate patients according to their overall survival (OS) (Figure 1).

### 2.2. Gene Expression Profile of Purified ALDH^bright^ and ALDH^low^ Cells, at Steady State and after Butein Treatment

**Figure 2:**
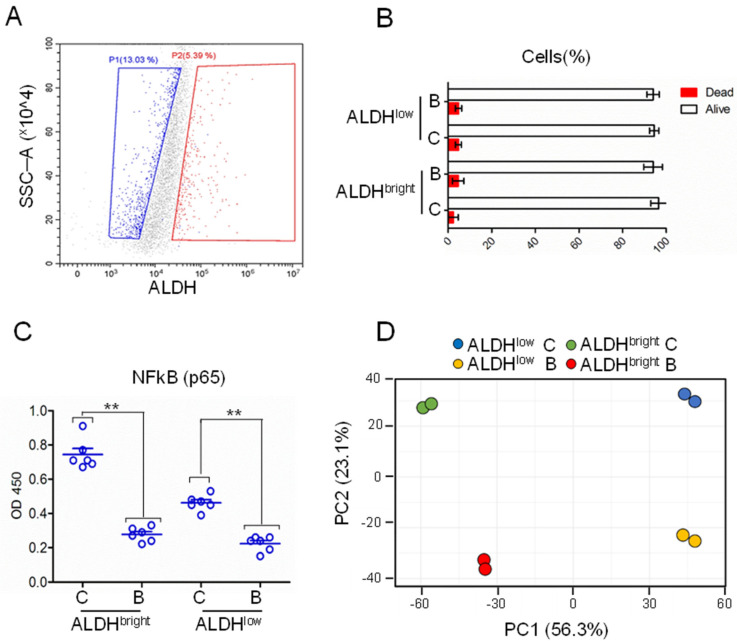
**Purification of mesothelioma ALDH^bright^ cell subpopulations**: (**A**) representative dot plots of NCI-H2595 MPM cells gated into ALDH^low^ (left) and ALDH^bright^ (right) cell subpopulations based on their ALDH activity. (**B**) Viability assay (incorporation of Sytox Blue dead cell stain) of the purified cell subpopulations treated with ctrl (**C**) (0.05% DMSO, 7 h) or with butein (**B**) (10 micromol/L, 7 h) after 2 h recovery from the FACS-based enrichment. No statistical difference was noted among all four treated samples in terms of percentage of dead cells. (**C**) Levels of p65 NFkB bound to a synthetic oligonucleotide from nuclear extracts of the purified cell subpopulations treated as indicated in (**C**) (*n* = 6). ELISA assay. Statistics: ** *p* < 0.01. ns = not significant. (**D**) PCA plots showing the distribution of the cell subpopulations considered in (**B**), based on the levels of 924 genes significantly modulated according to ALDH status and treatment.

The latter observation prompted us to purify, by means of FACS-sorting, the MPM cells expressing high levels of aldehyde dehydrogenase activity (ALDH) to interrogate their gene expression profile. Logarithmically grown NCI-H2595 mesothelioma cells were FACS sorted based on the levels of ALDH (ALDH^bright^ or ALDH^low^) (Figure 2A). Cells were allowed to recover from the sorting procedure for 2 h and treated either with butein or with 0.05% DMSO (ctrl) for 7 h (*n* = 2). After 7 h of treatment, no significant differences in cell death could be detected among control (C) and butein (B)-treated samples (*p* < 0.05) (Figure 2B). On the other hand, ELISA showed reduced binding of the p65 subunit of NFkB in the butein-treated samples, revealing effective target engagement by butein, which is a known NFkB inhibitor (Figure 2C).

### 2.3. Analyzing the Gene Expression Profile of ALDH^bright^ and ALDH^low^ Cell Subpopulations

When analyzing the results of the Affymetrix gene expression profiling, we adopted a linear modeling approach to identify genes that changed with respect to ALDH status (high vs. low), treatment (DMSO vs. butein treatment), or the interaction of the two, across all eight samples (four duplicate samples) (Tables S1–S4). We identified 924 genes whose differential modulation according to the ALDH: treatment status met high statistical significance (Benjamini–Hochberg false discovery rate (FDR)-corrected: *p* ≤ 0.25) (Table S5).

In Figure 2D, a plot was made of PC1 vs. PC2 and this showed strong separation between all four groups of samples. In agreement with their different biological features, the ALDH^low^ cells separated strongly from the ALDH^bright^ cells on the PC1 (56.3% of the total variance) (Figure 2D). The samples also separated in an orthogonal direction by butein treatment (PC2 23.1% of the total variance) (Figure 2D). This suggested that these two variables, namely high ALDH activity and butein treatment, were associated with strong and independent gene expression changes.

### 2.4. The MPM ALDH^bright^ Cells Exhibited Enrichment for NFkB-Related Pathways

**Figure 3:**
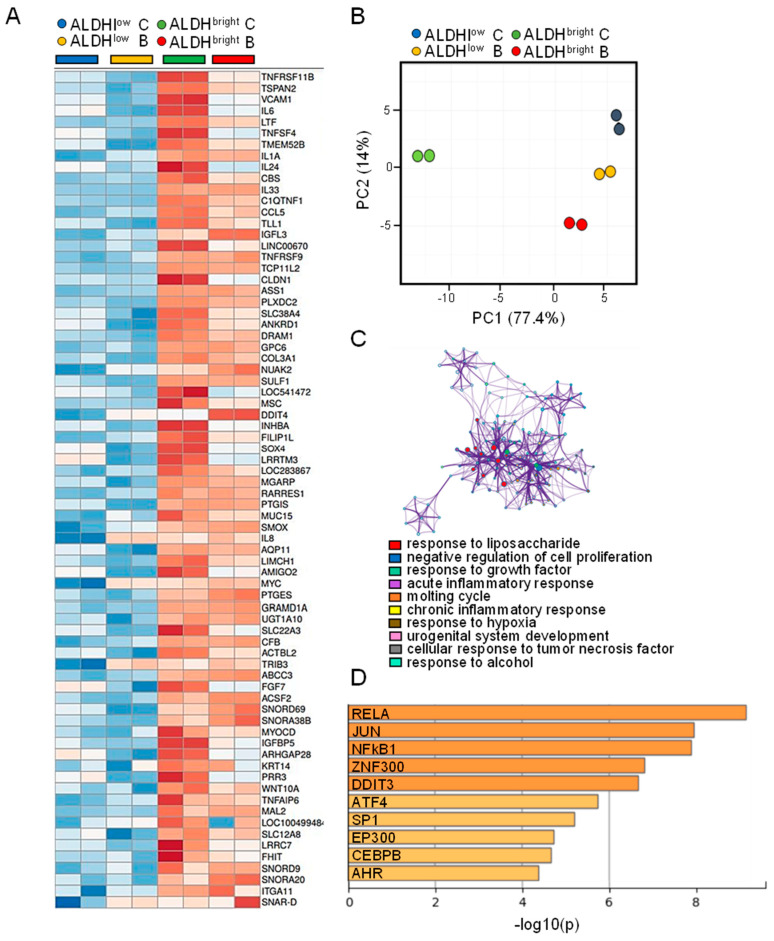
**Analysis of the genes enriched in ALDH^bright^ cells:** (**A**) representative heat map of the genes significantly enriched in the ALDH^bright^ cells (when compared to ALDH^low^ cells). Log2 (expression). *p* < 0.05, ≥ two-fold enriched mRNAs. (**B**) PCA plot showing the distribution of the cell subpopulations based on the levels of expression of the genes in (**A**). (**C**) Upper panel. The top 10 enriched GO terms between the groups with *p* < 0.05 were considered as enriched. The protein–protein interaction network: each term is represented by a circle node, with size proportional to the number of input genes. Lower panel. List of the most significantly enriched pathways as from upper panel. (**D**) Histogram showing the most significantly enriched binding sites (*p* < 0.05) within the promoters of the analyzed genes.

Within this experimental setting, we first focused on the genes significantly enriched by more than two-fold in the ctrl-treated ALDH^bright^ cells as compared to the ctrl-treated ALDH^low^ ones (Figure 3A). PCA analysis of this gene subset (*n* = 76) revealed that those genes were capable of differentiating the ALDH^bright^ cells from all the other experimental samples, on the main PC (PC1 77.4% of the main variance) (*p* < 0.05) (Figure 3B). Pathway analysis revealed enrichment for NFkB related pathways, such a LPS response, acute inflammation, chronic inflammation, in addition to pathways involved in negative regulation of cell proliferation (Figure 3C). These results confirmed our and others’ observations regarding the relevance of NFkB pathways for the maintenance of the chemoresistant ALDH^bright^ cells (see discussion). Additionally, other pathways related to wound repair and negative control of proliferation were enriched in the selected cluster; again matching the possibility that the ALDH^bright^ cells may represent a substantially different cell subpopulation compared to the ALDH^low^ counterparts (Figure 3C). Promoter enrichment analysis revealed a significant enrichment, within the analyzed gene promoters, for NFKB1 and RelA binding sites, thus supporting the previous observation based on the pathway analysis (Figure 3D). Further, we also found enrichment for DDIT3 binding sites (Figure 3D), supporting the existence of an NFkB-STAT3-DDIT3 axis controlling chemoresistance and ALDH^bright^ cell number in MPM [24].

### 2.5. The mRNAs Downregulated More Deeply in the ALDH^bright^ Cells by Butein Were Related to DNA Replication and Repair Functions

**Figure 4:**
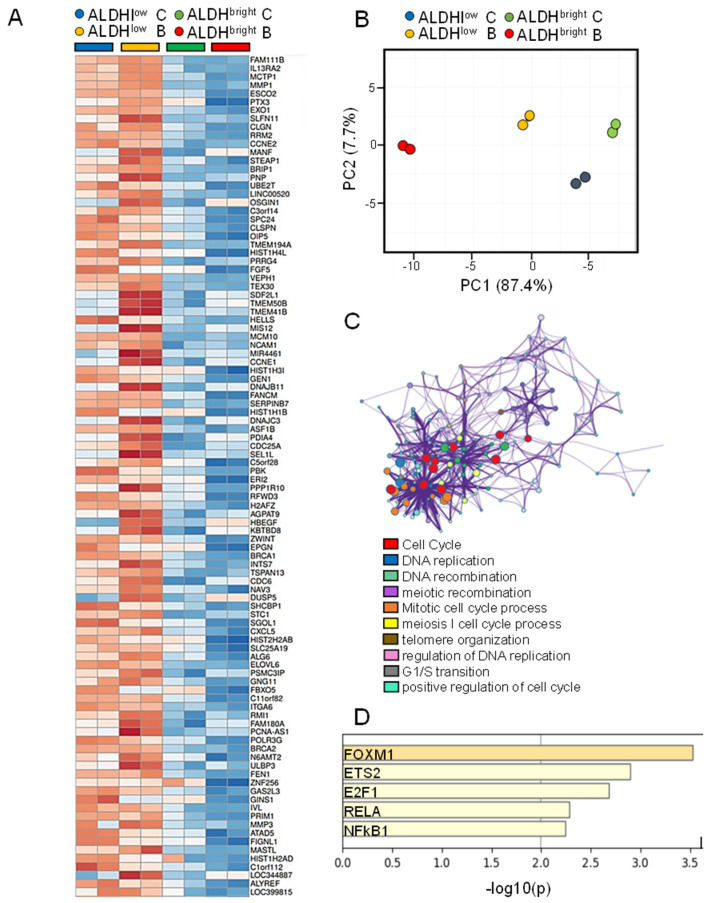
**Butein treatment dramatically affected the profile of ALDH^bright^ cells and ALDH^low^ cells**: (**A**) representative heat map of the genes significantly modulated more in the ALDH^bright^ cells (when compared to ALDH^low^ cells) after treatment with butein (**B**) for 7 h. log2 expression. *p* < 0.05, ≥two-fold modulated mRNAs between butein-treated ALDH^bright^ vs. ALDH^low^ cells. (**B**) PCA plot showing the distribution of the cell subpopulations based on the levels of expression of the genes considered in (**A**). (**C**) Upper panel. The top 10 enriched GO terms between the groups with *p* < 0.05 are shown. The protein–protein interaction network: each term is represented by a circle node, its size is proportional to the number of input genes. Lower panel. List of the most significantly enriched pathways as from upper panel. (**D**) Histogram showing the most significantly enriched binding sites (*p* < 0.05) within the promoters of the analyzed genes.

We subsequently focused on the genes whose expression was little to unchanged in the ALDH^bright^ cells as opposed to the ALDH^low^ cells but whose magnitude of downregulation by butein was higher in the ALDH^bright^ vs. ALDH^low^ cell (≥2-fold) (Figure 4A). PCA analysis evidenced the profound effect of butein treatment on the distribution of both ALDH^bright^ and ALDH^low^ samples (Figure 4B). The effect of butein was more pronounced in the ALDH^bright^ cells, as shown by the increased shift on the PC1 when compared to their ALDH^low^ counterparts (Figure S2). Pathway analysis revealed an enrichment for DNA damage repair pathways with a high enrichment score (Figure 4C). In detail, we found enrichment for cell cycle progression functions, DNA repair, DNA recombination and cellular response to radiations (Figure 4C). Interference with those pathways may underlie some of the chemo-sensitizing and anticancer actions of butein, including its ability to work as a Xanthine Oxidase inhibitor [34,35] (see discussion below). Promoter analysis of the target genes revealed enrichment for FOXM1, E2F and ETS2 (Figure 4D). FOXM1 drives the transcription of DNA damage sensors [36] and E2F1 interacts with DNA damage repair proteins at the foci of DNA damage and increases expression of DNA repair factors [37]. Finally, in coupling with the nature of the compound to act as an NFkB inhibitor [38,39], we found overrepresentation of NFkB1 and RelA binding sites in the promoter of the analyzed genes (Figure 4D). Altogether, we observed pathway-enrichment and promoter-enrichment features concordant with the mechanism of action of the drug and supportive of its chemo-sensitizing effects (see discussion below, please).

### 2.6. mRNAs Enriched in the ALDH^bright^ Cells May Have Prognostic Potential

**Figure 5:**
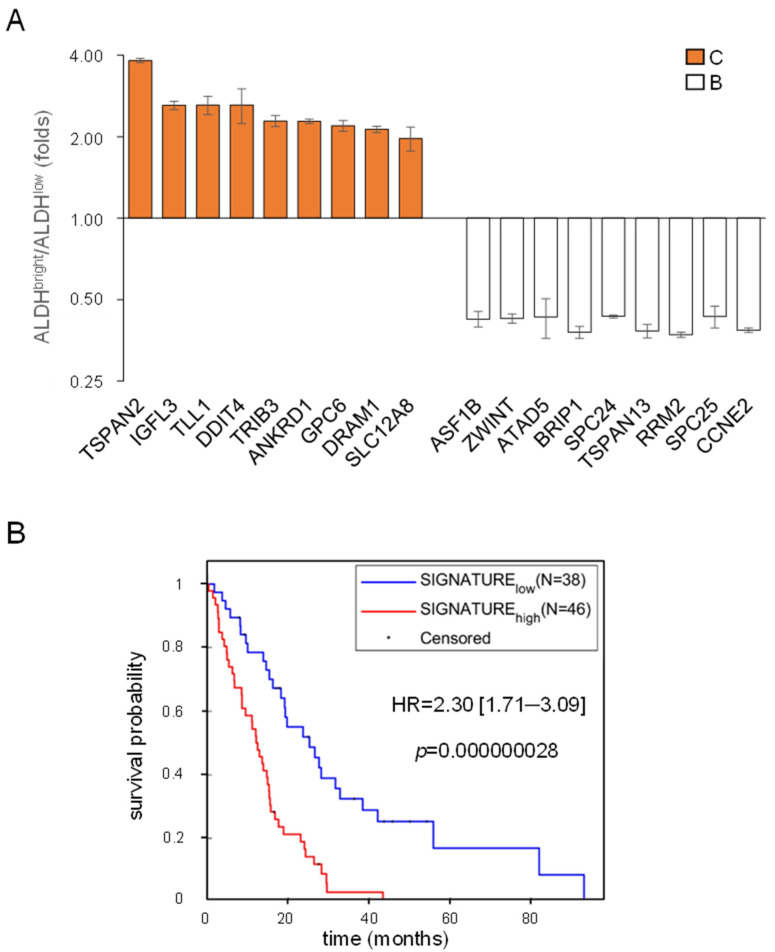
**Identification of an eighteen-gene signature endowed with prognostic significance:** (**A**) Histogram bars showing the levels of expression of 18 significantly modulated genes selected among the ALDH^bright^-enriched and those more deeply modulated by butein in the ALDH^bright^ cells (Figure 2 and Figure 3). Folds over ALDH^low^ cells. (**B**) Kaplan–Meier plot showing the distribution of 84 MPM patients from TGCA based on the mean levels of expression of the eighteen genes. The *p*- and HR values are reported.

We have shown that the expression of ALDH1A3 is prognostically relevant in MPM (Figure 1), and the MPM ALDH^bright^ cells predominantly expressed the ALDH1A3 isoform [24,33]. We also showed that butein abated the ALDH^bright^ cell number, at least partially through inhibiting a NFKB-STAT3-DDIT3 pathway [24]. Thus, we hypothesized that genes enriched in the ALDH^bright^ cells and those more downregulated, in the latter cells, by butein (as compared to their ALDH^low^ counterparts), may be endowed with prognostic potential. Among those mentioned genes, we selected, by the “leave one out” method, those that could represent a prognostic signature. This attempt was successful and allowed us to select an eighteen-gene signature, reported in Figure 5A.

We evaluated the mean signal of the selected eighteen genes, and the patients with high and low signals were defined by considering the positive and negative z-score values; by doing so, the eighteen-gene set exhibited high prognostic potential in terms of overall survival (OS) (*n* = 84) (Figure 5B). There was a median difference of almost 60 months when comparing patients with high signature levels to those with lower signature levels (Figure 5B).

Finally, we aimed at verifying whether performing such analysis in purified ALDH^bright^ cells bearing high levels of the prognostic ALDH1A3 mRNA [24] could have increased the sensitivity toward prognostically relevant mRNAs. To do so, we evaluated the levels of the eighteen mRNAs comprising the identified signature (Figure 5A) in a human MPM dataset, composed of 40 MPM and 9 normal samples, including five normal pleura specimens (GSE2549) (Figure S3). This revealed that, among the six genes that reached statistical significance (among the fifteen detectable), only two (TSPAN13, DDIT4) out of the eighteen genes were significantly modulated (*p* < 0.05), in a way coherent with what was observed in the ALDH^bright^ cells. On the other hand, SLC12A8, TLL1, TSPAN2 and RRM2 were oppositely modulated in the mentioned dataset, as compared to the ALDH^bright^ cells (Figure S3). This may indicate that using purified cell subpopulations as starting material may increase the chance of identifying prognostically relevant genes.

## 3. Discussion

Here, we reported the gene expression profiling of FACS-sorted MPM ALDH^bright^ cells from an MPM representative cell line. This follows a series of observations made by others and us in several experimental settings, suggesting that the ALDH^bright^ cells may mediate chemoresistance [22,33,40,41]. To date and to the best of our knowledge, a full gene expression profiling of FACS-sorted ALDH^bright^ cells is unprecedented in MPM. We have found that a significant number of genes whose levels of expression were enriched in the ALDH^bright^ cells as opposed to their ALDH^low^ counterparts were endowed with prognostic potential. The fact that a prognostic eighteen-gene signature could be derived from purified ALDH^bright^ cells further proves that the ALDH^bright^ cells in MPM may represent a functionally relevant cell subpopulation in the progression of the disease.

In more detail, we found, among the top modulated genes among ALDH^bright^ and ALDH^low^ cells, that there was a relative enrichment for NFkB target genes, matching the evidence that a short treatment with butein deeply modulated the NFkB-related pathways. This follows our and others’ observations pointing to the role of NFkB in the therapy-instigated emergence of ALDH^bright^ cells [24,42].

We have also analyzed the transcriptional changes taking place in cells treated with butein, which we have shown counteract to potentiate the effect of chemotherapy in vitro and in vivo [24]. Butein has pleiotropic anticancer effects; it has been shown to hit the PI3K/Akt-NFκB and the ATM-Chk1/2-Cdc25c-cdc2/cyclin B axes by inhibiting ROS generation [43,44]. Butein can enhance the TRAIL-induced apoptosis by activating the ERK-Sp1 pathway [45] and was shown to interfere with the telomerase activity and to downregulate c-MYC [46]. It is likely that, in our experimental conditions, butein acted at multiple points. This includes the NFkB/STAT3 pathway, whose contribution to the ALDH^bright^ cell emergence is pivotal. In fact, we have shown that DDIT3 upregulation, because of NFKB-STAT3 inhibition, mediated the increase in chemoresistant cells in pemetrexed-treated MPM samples [26]. We found that the levels of DDIT3 were increased in butein-treated ALDH^bright^ cells, thereby providing additional validation to our recent observations [24]. When analyzing the genes that were more deeply downregulated (>two folds ALDH^bright^ vs. ALDH^low^) in the ALDH^bright^ cells after butein administration, we found several pathways involved in DNA replication and repair. This echoes a preferential targeting of ALDH^bright^ cells by butein (Figure 1D) and the chemo-sensitizing effect observed in vitro, ex vivo and in vivo [24,26,44]. Butein may also function as a Xanthine Oxidase inhibitor when administered in a micromolar range [34,35]. Thus, it is possible that such an additional mechanism may mediate chemo-sensitization in MPM cells as well.

A limitation of this work is that we did not take in consideration, in our analysis, the distinction between epithelioid, biphasic and sarcomatoid MPMs by obtaining the gene expression data of corresponding cell lines. Those three main MPM histotypes bear profound difference in terms of gene expression profile, with prognostic implications [47]. Considering that the MPM cell line (NCI-H2595) used in this study was derived from a mainly epithelioid specimen [48], it is notable that the eighteen-gene signature appeared to perform equally well for both epithelioid and biphasic/sarcomatoid MPM subpopulations contained in the TGCA (data not shown). Therefore, this may suggest that the identified signature could be effective in both epithelioid and biphasic/sarcomatoid patients, despite having been derived from a predominantly epithelioid cells line. This may be due to the specific biological features of the MPM ALDH^bright^ cells and matches the recent evidence for the existence of a subpopulation of breast tissue-derived ALDH^bright^ cells that simultaneously express epithelial and mesenchymal markers [49]. This observation also meets the most current picture of MPM classification, framing MPMs as composed of a dynamic spectrum of cell subpopulations, going from epithelioid to sarcomatoid [50], possibly in response to tumor stage and treatment-specific adaptive conditions.

What ALDH^bright^ cells represent is still partially elusive. Beside the expression of ALDH1A3 and its modulation by butein, which leads to chemo-sensitizing effects [24], it is clear from our and others’ studies that this latter cell subpopulation is endowed with specific biological identity. Their increased number, in vitro and in vivo, upon chemotherapy-induced stress is noteworthy. Further, the prognostic potential of ALDH1A3 shown here and the enrichment of the ALDH^bright^ gene expression profile for pluripotency pathways and for the NFkB-related pathways may further contribute the idea that the ALDH^bright^ cells represent an intermediate cell subpopulation capable of conferring chemoresistance. This matches what we and others have shown about the ability of ALDH^bright^ cells to generate ALDH^low^ cells, acting as at least bipotent progenitors and their enrichment for EMT genes [18,33,41,51].

Intra-tumor heterogeneity is only partially described in MPM, in part due to the use of omics approaches mainly directed toward bulk tumor samples [52]. Malignant pleural mesotheliomas are composed of multiple sub-clones with variable frequency [52,53,54]. This work provides a suggestion that investigating intra-tumor heterogeneity through isolating and characterizing specific cell subpopulations may provide clinically useful results. Most of the genes whose modulation in the chemoresistant cells has been unveiled by our study would otherwise have escaped the analysis of unfractionated samples. In fact, we found that only two (TSPAN13, DDIT4) of the eighteen genes identified as composing the prognostic signature were significantly and concordantly modulated in an independent set obtained from bulk samples (GSE2549) (Figure S3). Two additional genes (TLL1, TSPAN2), whose levels were higher in ALDH^bright^ cells as compared to the ALDH^low^ cells (Figure 3), exhibited opposite modulation in the normal as compared to the neoplastic pleura (Figure S3). This may be due to the low frequency of ALDH^bright^ cells within the unfractionated cell culture [24] and to the relative enrichment consequent to the FACS sorting procedure. On the other hand, the RRM2 exhibited higher levels in the MPM tumors and lower levels in the ALDH^bright^ cells, respectively (Figure S3).

The optimized eighteen-gene signature may evoke a certain tumor property, such as belonging to a novel subgroup or putative resistance to pemetrexed treatment, or may even represent a marker for the presence of more aggressive cell subpopulations (comprising the ALDH^bright^ cells). We also found that patients bearing high mRNA levels of ALDH1A3 mRNA fared worse, after radio- and or chemotherapy, than those exhibiting lower ALDH1A3 levels. However, the number of patients treated with Tx/Rx after diagnosis was too low to allow robust conclusions (*n* = 13 and *n* = 9, respectively, *p*: 0.19) (unpublished observation). This certainly prompts for further, future investigations of potential translational relevance.

## 4. Materials and Methods

### 4.1. Cell Lines and Treatments

The human MPM cell lines NCI-H2595 were from Prof. Harvey Pass lab, New York, NY, USA. The cells were cultured as monolayers at 37 °C and 5% CO_2_ in DMEM/F12 + GLUTAMAX supplemented with 10% non-heat-inactivated FBS (fetal bovine serum; Invitrogen-Gibco, Carlsbad, CA, USA). The cell line was in house tested for mycoplasma contamination by using a commercially available PCR-based assay (R&D Systems, Minneapolis, MN, USA). Butein (Santa Cruz Biotechnology, Dallas, TX, USA) was dissolved in DMSO according to the manufacturer’s instructions. Ctrl was 0.05% DMSO, accordingly.

### 4.2. ALDH Detection

ALDH activity was assessed by flow cytometry in MPM cell line subsets using an ALDEFLUOR kit (Stem Cell Technologies Vancouver, BC, Canada) in accordance with the manufacturer’s instructions. Briefly, the cells were incubated with BODIPY aminoacetaldehyde (BAAA), which is converted into a fluorescent molecule (BODIPY aminoacetate) in the cytoplasm. Specificity of the fluorescence was shown using the specific ALDH inhibitor diethylaminobenzaldehyde (DEAB). To eliminate dead cells, cells were stained with viability stain Sytox-Red (Life Technologies Inc., Grand Island, NY, USA). Cell populations were identified using a CytoFLEX flow cytometer (Beckman Coulter Life Sciences, Indianapolis, IN, USA). Distinct Aldefluor-positive and Aldefluor-negative populations were revealed after excluding debris and dead cells quantitated by Sytox blue staining (Thermo Fischer, Waltham, MA, USA) Analysis was performed by using the CytExpert software (Beckman Coulter Life Sciences, Indianapolis, IN, USA).

### 4.3. FACS-Based Purification of ALDH^bright^ and ALDH^low^ Cells

Cells were gently detached with Accutase (Stem Cell Technologies Vancouver, BC, V6A 1B6, Canada), filtered through a 40 μM mesh to obtain a single-cell suspension and were treated with BAAA or DEAB on ice and then incubated for 30 min at 37 °C, 5% CO_2_. Cell sorting was performed with a BD ARIA II (BD Biosciences, Franklin Lakes, NJ, USA). Gates were drawn to exclude >97% of non-specific staining (based on the background staining of the DEAB treated cells) and to exclude dead/apoptotic cells as indicated before. Purity of the enriched subpopulations was 95–97%, as assessed by the ALDH assay within 6 h after sorting.

Detection of DNA-bound p65 by ELISA. For detecting of NFkB p65 levels, an ELISA-based assay (NFkB p65 Transcription Factor Assay Kit AB133112, ABCAM, Cambridge, UK) was used, according to the manufacturer’s instructions. For extract preparation, a Nuclear Extraction Kit, ab113474, ABCAM, Cambridge, UK) was used, according to the manufacturer’s instructions.

### 4.4. RNA Extraction and Analysis

Total RNA was extracted using the Trizol Reagent (Life Technologies, Monza, Italy) and RNA quality was checked by means of a 2100 Bioanalyzer system (Agilent Technologies, Santa Clara, CA, USA).

### 4.5. Q-RTPCR

The first-strand cDNA was synthesized according to the manufacturer’s instructions (M-MLV RT kit, Invitrogen, Waltham, MA, USA). Gene expression was measured by real-time PCR using the SYBR-Green assay (Cell Signaling Technology, Inc., Danvers, MA, USA) on a 7900HT instrument (Thermo Fisher Scientific, Waltham, MA, USA). Beta-actin was used as reference control. All the primers were commercially available (Human qPCR Primer Pair kit, OriGene Technologies, Inc., Rockville, MD, USA).

### 4.6. Microarray Analysis

Affymetrix hybridizations were performed according to the manufacturer’s instructions. Affymetrix GeneChip Human Gene 2.0 ST CEL files were normalized to produce gene-level expression values using the implementation of the Robust Multiarray Average (RMA) in the Affymetrix package (version 1.36.1) included within in the Bioconductor software suite and an Entrez gene-specific probe set mapping from the Molecular and Behavioral Neuroscience Institute (Brainarray) at the University of Michigan. Array quality was assessed by computing Relative Log Expression (RLE) and Normalized Unscaled Standard Error (NUSE) using the AFFYPLM Bioconductor package (version 1.34.0). Differential gene expression was assessed using the moderated (empirical Bayesian) *t*-test implemented in the Limma package (i.e., creating simple linear models with lmFit, followed by empirical Bayesian adjustment with eBayes). Correction for multiple hypothesis testing was accomplished using the Benjamini–Hochberg false discovery rate (FDR). All microarray analyses were performed using the R environment for statistical computing.

### 4.7. Differential Gene Expression Statistical Analysis

For each effect (ALDH, treatment and ALDH-treatment), *t*-tests were performed on the corresponding coefficient of the linear model to obtain a t-statistic and *p*-value for each gene (Table S1). A “moderated” *t*-test was used, which leveraged information from all of the genes on the array to increase statistical power over a standard two-sample Student’s *t*-test. Benjamini–Hochberg false discovery rate (FDR) correction was then used to obtain FDR-corrected *p* values (*q* values), representing the probability that a given result is a false positive based on the distribution of all *p*-values (Table S2). The main effects of ALDH status and butein treatment were assessed using a linear model of the form: expression~ALDH + treatment and the interaction effect of ALDH status and treatment was assessed with a linear model of the form expression~ALDH + treatment + ALDH:treatment. For each effect (ALDH, treatment, and ALDH:treatment), *t*-tests were performed on the corresponding coefficient of the linear model to obtain a t-statistic and *p*-value for each gene. A “moderated” *t*-test was also used to leverage information from all of the genes on the array to increase statistical power. Pairwise *t*-tests were performed between treatment groups within each ALDH group (Table S3) and between ALDH groups within each treatment group (DMSO vs. butein) (Table S4).

### 4.8. Validation of the mRNA Expression

To validate the gene expression data, we performed QRT-PCR analysis of 34 genes belonging to those enriched in the ALDH^bright^ cells and to those more downregulated by butein in the ALDH^bright^ cells. This revealed a high concordance with the normalized Affymetrix intensity values, and this is shown by linear correlation analysis in Figure S2 (r square: 0.7943).

### 4.9. Principal Component Analysis (PCA)

For creating PCA plots, Clustvis was employed (https://biit.cs.ut.ee/clustvis/) accessed on 9 September 2021 [55].

### 4.10. Pathway Enrichment Analysis

Metascape [56] was used to extract comprehensive biological information associated with large candidate gene lists. Gene ontology (GO) analysis of the target genes of the differentially expressed mRNAs was performed in this study. By bioinformatics analysis, GO terms were selected from the significantly enriched gene sets (*p* < 0.05). The top 10 enriched GO terms between the groups with *p* < 0.05 were considered significantly enriched. The protein–protein interaction network analysis was performed in a way that each term is represented by a circle node, where its size is proportional to the number of input genes falling into that term, and its color represent its cluster identity (i.e., nodes of the same color belong to the same cluster). Promoter enrichment analysis was performed with TRRUST [57].

### 4.11. Identification of a Prognostic MPM Signature

The performance of minimized signatures was validated by “leave-one-out” cross validation. Normalized gene expression was downloaded from Broad Institute TCGA Genome Data Analysis Center (http://gdac.broadinstitute.org/): Firehose stddata__2016_01_28. Broad Institute of MIT and Harvard. https://doi.org/10.7908/C11G0KM9 (accessed on 9 September 2020). The clinical information was obtained from cBioPortal https://www.cbioportal.org/) (accessed on 20 September 2021). Overall survival (OS) was calculated by using Kaplan–Meier analysis and the log-rank test was used to assess differences between curves. A Cox proportional-hazards regression model was built to evaluate the effect of the clinical variables on survival analysis. For the gene’s signature we evaluated the mean signal of the selected genes and patients with high and low signal were defined by considering the positive and negative z-score values, respectively. The analyses were conducted with Matlab R2020b. The eighteen genes composing the signature were studied in a human dataset comprising composed of 40 MPM and 9 normal samples, including five normal pleura specimens (GSE2549).

## Data Availability

All data generated or analyzed during this study are included in this published article [and its supplementary information files].

## Supplementary Materials

The following are available online at https://www.mdpi.com/article/10.3390/ijms222112071/s1.

## Abbreviations

ALDH	Aldehyde Dehydrogenase
MPM	Malignant Pleural Mesothelioma
ELISA	Enzyme-linked immunosorbent assay
NFkB	nuclear factor of kappa light polypeptide gene enhancer in B-cells
CSC	Cancer Stem Cell
EMT	Epithelial to Mesenchymal Transition
FACS	Fluorescence Activated Cell Sorting
