# Supplementary material for: Insights into Intra-Tumoral Heterogeneity: Transcriptional Profiling of Chemoresistant MPM Cell Subpopulations Reveals Involvement of NFkB and DNA Repair Pathways and Contributes a Prognostic Signature
Source: Int J Mol Sci. 2021 Nov 8;22(21):12071. doi: 10.3390/ijms222112071 (PMC8585077; doi:10.3390/ijms222112071)
Supplement: Supplementary file 1 [file ijms-22-12071-s001.zip › Legends to supplementary figures.pdf]

Legends to supplementary figures

Figure S1. Validation of the gene expression results. A. Histogram reporting the Q-RTPCR analysis of 34 representative transcripts from the gene expression profile obtained. B. Correlation analysis between the Affymetrix normalized intensity values (x-axis) and the Q-RTPCR expression data (y-axis), all expressed as folds over ctrl (FOC). The *r* square value is indicated.

Figures S2. Box plot showing the relative distance on the PC1 of the selected cell subpopulations. Statistics: * $p<0.05$; ** $p< 0.01$.

Figure S3. The eighteen-gene signature is only partially identifiable when analyzing bulk tumors. Box plot showing the levels of the fifteen detectable out of eighteen genes identified in this work, as assessed in the GSE2549 human MPM dataset which contains 40 human MPM tumor specimens and 9 normal specimens of which four are normal lung and five are normal pleura (https://www.ncbi.nlm.nih.gov/geo/query/acc.cgi?acc=GSE2549). The *p* value is reported.
