# Supplementary material for: Insights into Intra-Tumoral Heterogeneity: Transcriptional Profiling of Chemoresistant MPM Cell Subpopulations Reveals Involvement of NFkB and DNA Repair Pathways and Contributes a Prognostic Signature
Source: Int J Mol Sci. 2021 Nov 8;22(21):12071. doi: 10.3390/ijms222112071 (PMC8585077; doi:10.3390/ijms222112071)
Supplement: Supplementary file 1 [file ijms-22-12071-s001.zip › Suppl. Figs.pptx]

## Slide 1
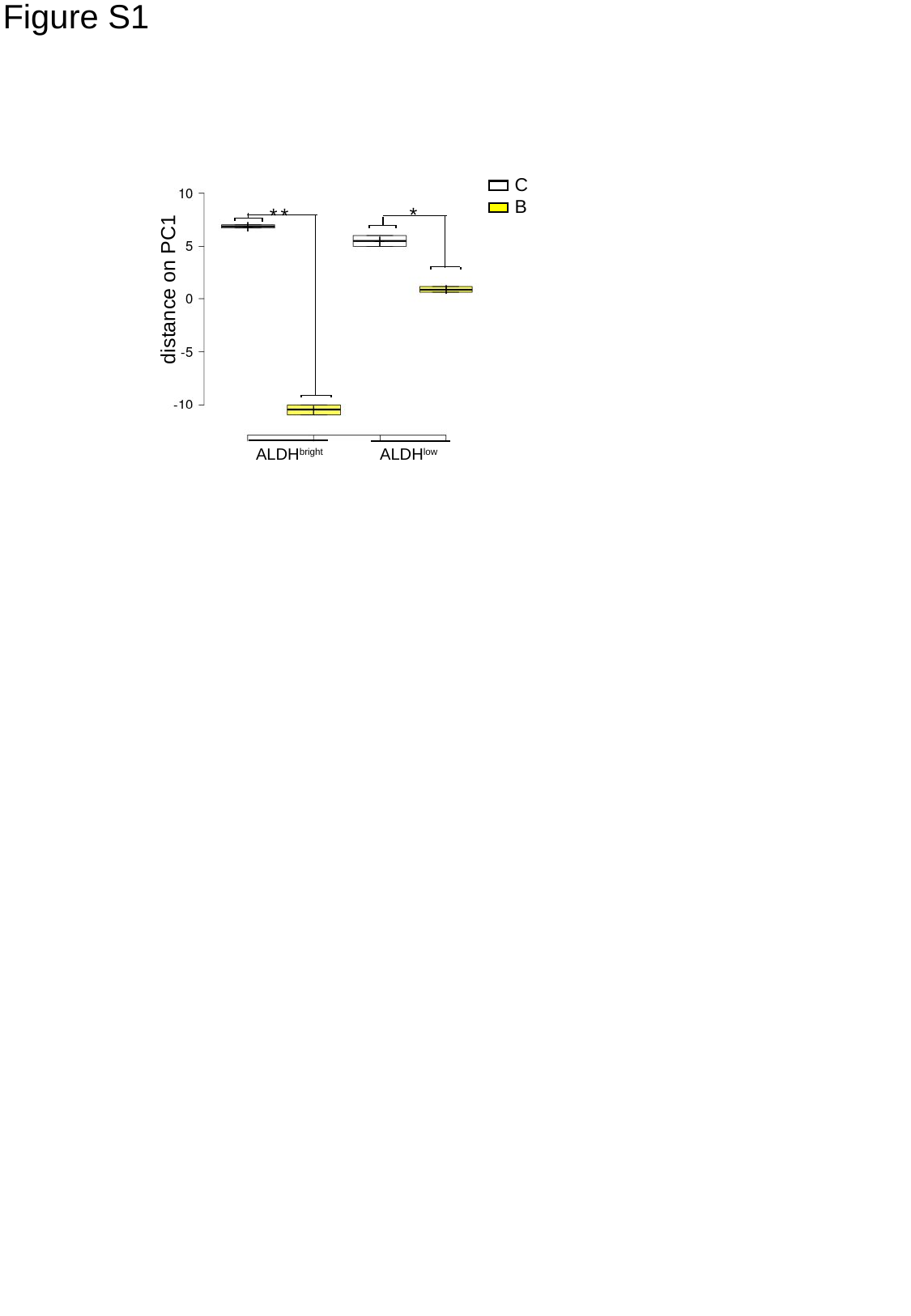

Figure S1
C
B
*
*
*
distance on PC1
ALDHbright
ALDHlow

## Slide 2
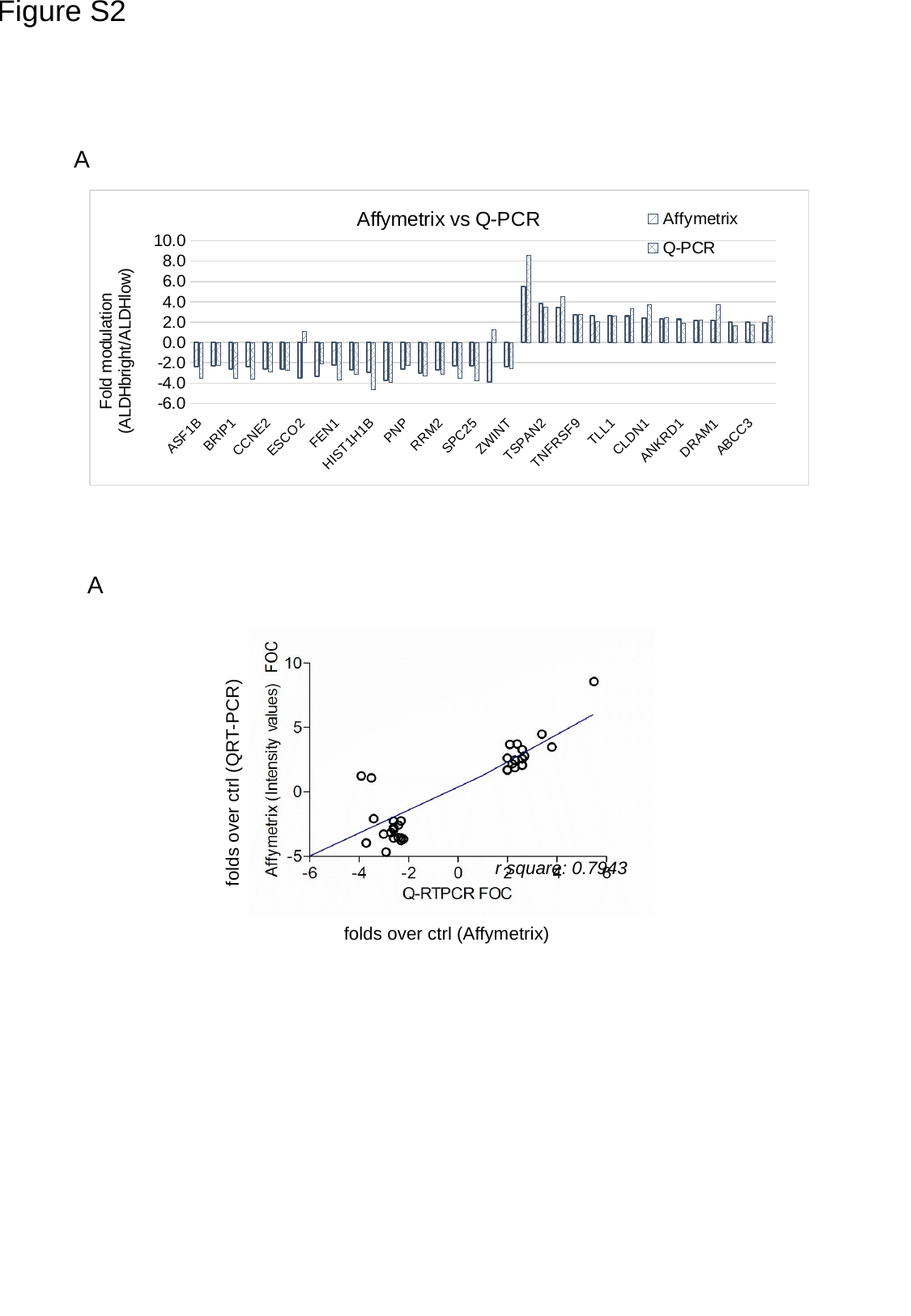

Figure S2
A
### Chart: Affymetrix vs Q-PCR
| Category | Affymetrix | Q-PCR |
|---|---|---|
| ASF1B | -2.36112992326091 | -3.5549999999999997 |
| ATAD5 | -2.33143420370523 | -2.255 |
| BRIP1 | -2.64439811503634 | -3.575 |
| CCNE1 | -2.34912129881574 | -3.5949999999999998 |
| CCNE2 | -2.59538086561273 | -2.925 |
| CLSPN | -2.58709371426566 | -2.7749999999999995 |
| ESCO2 | -3.47885634968387 | 1.08 |
| EXO1 | -3.36900809741863 | -2.075 |
| FEN1 | -2.19263571536253 | -3.675 |
| GPR75 | -2.69142146392669 | -3.175 |
| HIST1H1B | -2.91864665324349 | -4.675000000000001 |
| MCTP1 | -3.7469321599971 | -3.975 |
| PNP | -2.60341277255072 | -2.2750000000000004 |
| PTX3 | -3.01326435579928 | -3.2749999999999995 |
| RRM2 | -2.69823004294683 | -3.175 |
| SPC24 | -2.30618898653111 | -3.575 |
| SPC25 | -2.30639967124853 | -3.7749999999999995 |
| ULBP3 | -3.87262236463125 | 1.23 |
| ZWINT | -2.35337278794649 | -2.575 |
| TNFRSF11B | 5.5077042482964 | 8.575 |
| TSPAN2 | 3.82337060653241 | 3.475 |
| TNFSF4 | 3.41656934558596 | 4.475 |
| TNFRSF9 | 2.6967153555568 | 2.775 |
| IGFL3 | 2.6122745398077 | 2.075 |
| TLL1 | 2.61205366521056 | 2.575 |
| DDIT4 | 2.59619419937923 | 3.275 |
| CLDN1 | 2.38976582115665 | 3.715 |
| TRIB3 | 2.28922341385142 | 2.475 |
| ANKRD1 | 2.27872950823643 | 1.875 |
| GPC6 | 2.19137114349871 | 2.175 |
| DRAM1 | 2.12723336440916 | 3.675 |
| AMIGO2 | 1.99855310535979 | 1.675 |
| ABCC3 | 1.99717361111338 | 1.715 |
| SLC12A8 | 1.95194985844272 | 2.615 |A
folds over ctrl (QRT-PCR)
r square: 0.7943
folds over ctrl (Affymetrix)

## Slide 3
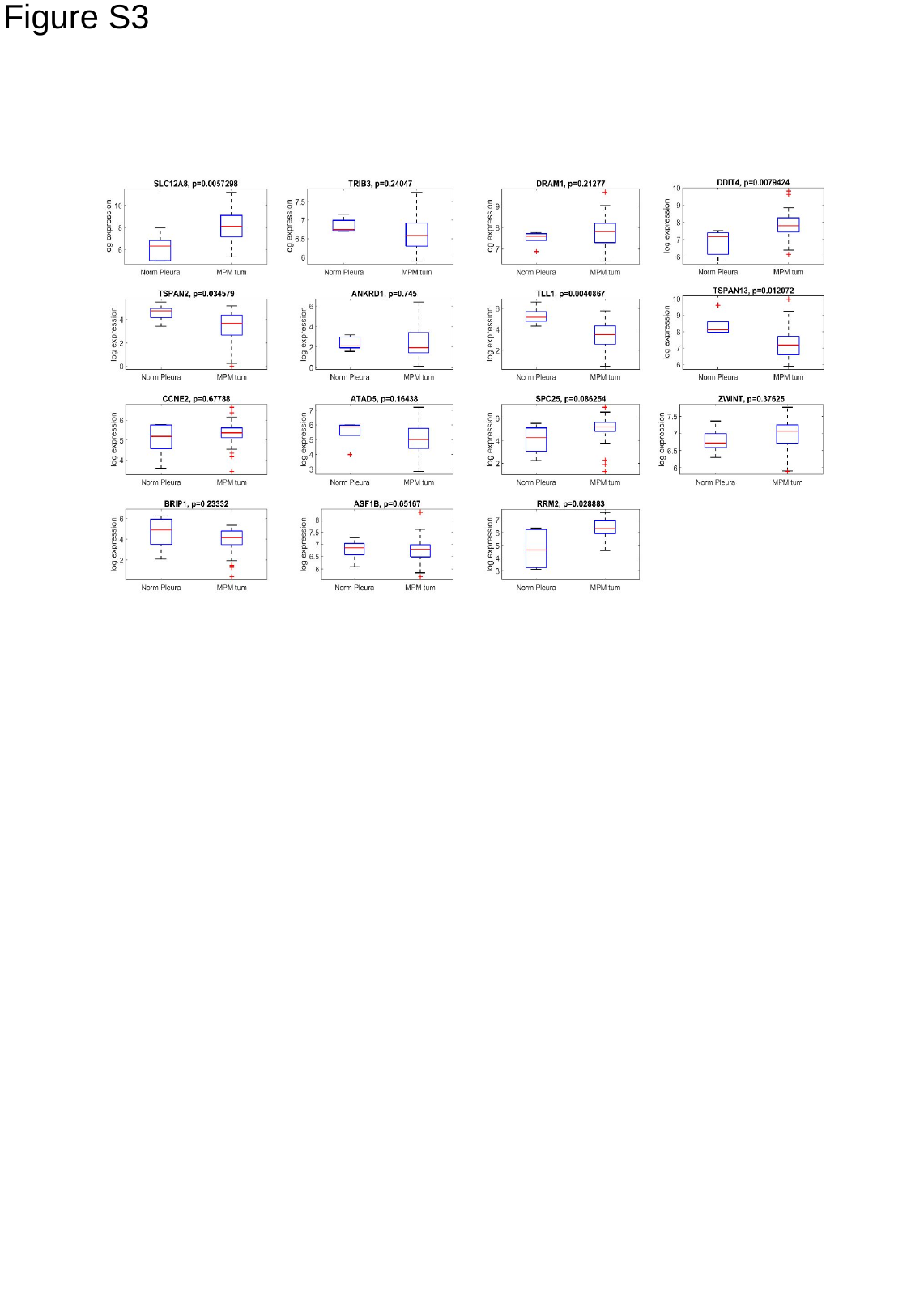

Figure S3
