# Supplementary material for: Insights into Intra-Tumoral Heterogeneity: Transcriptional Profiling of Chemoresistant MPM Cell Subpopulations Reveals Involvement of NFkB and DNA Repair Pathways and Contributes a Prognostic Signature
Source: Int J Mol Sci. 2021 Nov 8;22(21):12071. doi: 10.3390/ijms222112071 (PMC8585077; doi:10.3390/ijms222112071)
Supplement: Supplementary file 1 [file ijms-22-12071-s001.zip › Suppl. Tables 1-4.pdf]

Table S1. Differentially expressed genes due to each effect. Table S1 below shows the number of genes with a p value below various thresholds, as well as the number of genes expected by chance at each threshold, for each effect. The left part of both tables summarizes the number of genes with a moderated p value below various thresholds; the right part of both tables below summarizes the FDR q values for the same comparisons

p threshold	expected	ALDH	Treatment	ALDH:treatment
0.05	1189	6428	6223	2841
0.01	238	3684	3552	939
0.005	119	2839	2812	615
0.001	24	1474	1507	199

Table S2. FDR q values for each effect.

q threshold	ALDH	treatment	ALDH:treatment
0.25	7552	7229	924
0.1	4669	4401	86
0.05	3128	3037	2
0.01	972	1100	0

Table S3. Summary of p and FDR q values between the butein- and ctrl-treated samples within each ALDH group. The left part of both tables summarizes the number of genes with a moderated p value below various thresholds; the right part of both tables below summarizes the FDR q values for the same comparisons

butein vs ctrl							
p threshold	expected	$ALDH^{low}$	$ALDH^{bright}$		q threshold	$ALDH^{low}$	$ALDH^{bright}$
0.05	1189	5428	4880		0.25	6103	4989
0.01	238	2472	2138		0.1	2568	1902
0.005	119	1745	1501		0.05	1191	824
0.001	24	704	594		0.01	37	0

Table S4. Summary of p and FDR q values between the $ALDH^{bright}$ and $ALDH^{low}$ cells within each treatment group. Moderated t tests: $ALDH^{bright}$ vs. $ALDH^{low}$, within each treatment type. The left part of both tables summarizes the number of genes with a moderated p value below various thresholds; the right part of both tables below summarizes the FDR q values for the same comparisons

$ALDH^{bright}$ vs $ALDH^{low}$						
**p threshold**	**expected**	**ctrl**	**butein**	**q threshold**	**ctrl**	**butein**
0.05	1189	4343	6180	0.25	3959	7694
0.01	238	1740	2798	0.1	1007	3294
0.005	119	1125	1931	0.05	7	1455
0.001	24	362	725	0.01	0	0

Table S5. Summary of mRNA expression data of the eighteen genes identified in this work wghen bulk, matched mesothelial vs mesothelioma specimens were analyzed.

